# Case report: a case of hypoparathyroidism–sensorineural deafness–renal dysplasia syndrome

**DOI:** 10.3389/fgene.2025.1501427

**Published:** 2025-04-22

**Authors:** Jinyan Yang, Yanjie Mei, Feifei Tang, Xinhong Guo, Yanhua Kong, Ying Deng

**Affiliations:** ^1^ Department of Endocrinology, Bozhou People's Hospital, Anhui Province, China; ^2^ Department of Endocrinology, The First Affiliated Hospital of Nanchang Medical University, Jiangxi, China

**Keywords:** *GATA3* gene, HDR syndrome, hypoparathyroidism, sensorineural deafness, renal dysplasia

## Abstract

This article reports a case of a young woman who was admitted to the hospital with “sudden convulsions for 3 h.” She was diagnosed with hypoparathyroidism and found to have sensorineural deafness and left renal agenesis. A diagnosis of hypoparathyroidism–sensorineural deafness–renal dysplasia (HDR) syndrome was established, and the patient was treated with calcium and active vitamin D. After 2 years of follow-up, her blood calcium levels continued to fluctuate significantly. Subsequently, a heterozygous variant in the *GATA3* gene (NM_001002295.2:c.404dup) was detected. According to the literature, patients with HDR syndrome require low doses of active vitamin D supplementation. Excessively high blood calcium levels should be avoided, and treatment should be individualized.

## Introduction

Hypoparathyroidism–sensorineural deafness–renal dysplasia syndrome (HDRS) is an dominant disorder caused by mutations in the *GATA3* gene and is characterized by the triad of hypoparathyroidism, sensorineural deafness, and renal dysplasia, which was first reported in 1977 by Barakat et al. HDRS is rare, and due to its frequent misdiagnosis and unclear prevalence, there are few domestic reports on HDRS and few studies on the relationship between the clinical phenotype and genotype heterogeneity in HDRS patients (Shu-yao, 2021). In this paper, we report a case of a patient with hypoparathyroidism, sensorineural deafness, and renal agenesis who was diagnosed with HDRS through GWAS, with the aim of enhancing the understanding of HDRS, reducing misdiagnosis, and improving therapeutic efficacy.

## Summary of medical records

The patient, a 25-year-old woman, was admitted to Bozhou People’s Hospital of Anhui Province on 21 August 2019 due to “sudden convulsions for 3 h.” During the seizure, the patient’s upper limbs were flexed and lower limbs were straightened, accompanied by rolling eyes, foaming at the mouth, unconsciousness, and inability to call out, which lasted for approximately 5 min and then relieved on its own, and the patient gradually became conscious, and the seizure occurred for once in total. The patient experienced a single episode of nausea and vomiting during the period, with emesis consisting of gastric contents. This episode was supposed to be related to “epilepsy,” and the patient was admitted to the neurology department. The patient was admitted to the gastroenterology department in September 2015 due to gastric discomfort and was found to have a blood calcium level of 1.19 mmol/L, with no self-consciousness or discomfort, and was not further diagnosed or treated. She was operated on 27 March 2019 due to breast nodules and was found to have a blood calcium level of 1.70 mmol/L (albumin of 47.2 g/L), with no self-consciousness or discomfort, and was not treated. Hearing abnormality was found for more than 10 years, particularly noticeable in the right ear. She had a history of cesarean section five years ago, a history of blood transfusion, a history of pregnancy and abortion, 1-0-2-1, and a history of alcohol consumption for 7 years, approximately 100 mL of white wine per day. She denied any family history of similar diseases. Additionally, her parents were not consanguineous.

Admission physical examination: temperature (T), 36.5°C; pulse (P), 67 times/min; respiratory rate (R), 19 times/min; blood pressure (BP), 93/59 mmHg. The patient was alert but showed poor spirit and intellectual disability, with clear speech. There was perioral and limb numbness, and no deformity of the skull was observed. There were no malformations in facial development, and there was no eyelid puffiness. Hearing impairment was noted, particularly in the right ear. Pure-tone audiometry revealed decreased binaural air and bone conductance hearing thresholds to approximately 50 dB, showing moderate sensorineural hearing loss. Dental development was normal; the neck was soft; thyroid was not enlarged; and examination of heart, lungs, and abdomen (−) showed no abnormalities. There were no malformations of the spine or extremities; there was no swelling in the bilateral lower limbs, and muscle strength was normal. All sensory functions were normal. Neurologic examination showed no significant abnormalities. Chvostek’s sign was positive, and Trousseau’s sign was positive.

Laboratory tests: blood routine, troponin I, brain natriuretic peptide (BNP), coagulation function, urine routine, fecal routine, liver and kidney function, blood lipids, glucose, glycosylated hemoglobin, blood sedimentation, and C-reactive protein (CRP) did not show any significant abnormality. Albumin: 35.6 g/L (reference value: 35.0–55.0 g/L), potassium: 3.71 mmol/L (reference value: 3.5–5.5 mmol/L), sodium: 137.6 mmol/L (reference value: 135–145 mmol/L), chloride: 96.5 mmol/L (reference value: 96–106 mmol/L), calcium: 1.50 mmol/L (reference value: 2.10–2.55 mmol/L), and carbon dioxide: 30.1 mmol/L. Review: calcium = 1.51 mmol/L, magnesium = 0.77 mmol/L, phosphorus = 1.79 mmol/L (reference value: 0.80–1.82 mmol/L), and 24-h urinary calcium = 3.63 mmol/d. Synchronized parathyroid hormone: 23.48 pg/mL (reference value: 0–80 pg/mL); 25 hydroxyvitamin D: 21.82 ng/mL (reference value: 30–100 ng/mL). Adrenocorticotropic hormone, cortisol, thyroid function and antibodies, rheumatoid factor, beta-microglobulin, immunoglobulin, anti-neutrophil cytoplasmic antibody, complement, IgG4, autoantibody 16, glutamic acid decarboxylase antibody, and insulin autoantibody were not found to be abnormal. Imaging: cranial computed tomography (CT) showed basal ganglia calcification. Electroencephalogram (EEG) topography showed that the basal rhythm was dominated by 10–12 Hz alpha waves, roughly symmetrical on both sides, with a slightly poor amplitude modulation, a little suppression of alpha waves in the eyes-open and eyes-closed test, and a small number of medium-amplitude slow waves in the deep respiration test in the various leads of the formation, suggesting a mild EEG abnormality. Electrocardiogram: sinus rhythm; normal electrocardiogram (QT interval: 424 ms). Cardiac ultrasound: small amount of tricuspid regurgitation. Thyroid and parathyroid color ultrasound: no obvious abnormality. Ultrasound: renal agenesis, left kidney, not explored; right kidney, no echo—limited calyceal dilatation possible. The patient’s past reproductive history was not documented at our hospital, and the patient and her family were asked whether there was any history of renal agenesis or relevant examinations such as abdominal ultrasound, and they reported no such findings. Neck CT scan + double phase enhancement: neck CT scan and enhancement showed no obvious abnormality. Peripheral blood chromosome examination showed no abnormality. Ophthalmologic examination showed no cataracts (see Table 1). The Chinese-Adult Wechsler Intelligence Scale score was 68. Diagnostic and therapeutic procedures: the patient’s blood calcium level was obviously low, with epileptic seizures, and she was given intravenous calcium gluconate, calcium carbonate (1.0 g/day), and osteotriol (0.75 ug/day), with a dynamic review of the blood calcium level and adjustment of the osteotriol dose. The dosage of the medication is shown in Table 2.

**TABLE 1:** Clinical features of our case of HDR and those reported in the literature.

Clinical manifestation	Frequency of occurrence in the literature (Tao et al., 2023)	The patient in this case showed
Hypoparathyroidism	93.82%	+
Seizures	33.53%	+
**Tetany**	17.37%	
Basal ganglia calcification	8.38%	+
Pause for breath	1.8%	
Cataracts	1.8%	
Intellectual disability	8.38%	+
Hearing impairment	98.9%	+
Kidney damage	74.7%	
Renal dysplasia	41.9%	
Renal agenesis	22.58%	+
Vesicoureteral reflux	18.55%	
Asymptomatic	37.13%	

**TABLE 2:** Patients’ blood calcium levels and dosage of calcium carbonate and calcitriol.

Date	Calcium (mmol/L)	Phosphorus (mmol/L)	Elemental calcium (g/d)	Calcitriol (ug/d)
2019-08-21	1.50	1.79	1.0	0.75
2019-08-24	2.16	1.76	1.0	0.50
2019-08-25	2.35	1.80	1.0	0.25
2019-08-26	2.50	1.98	1.0	Stopped medication

**FIGURE 1:**
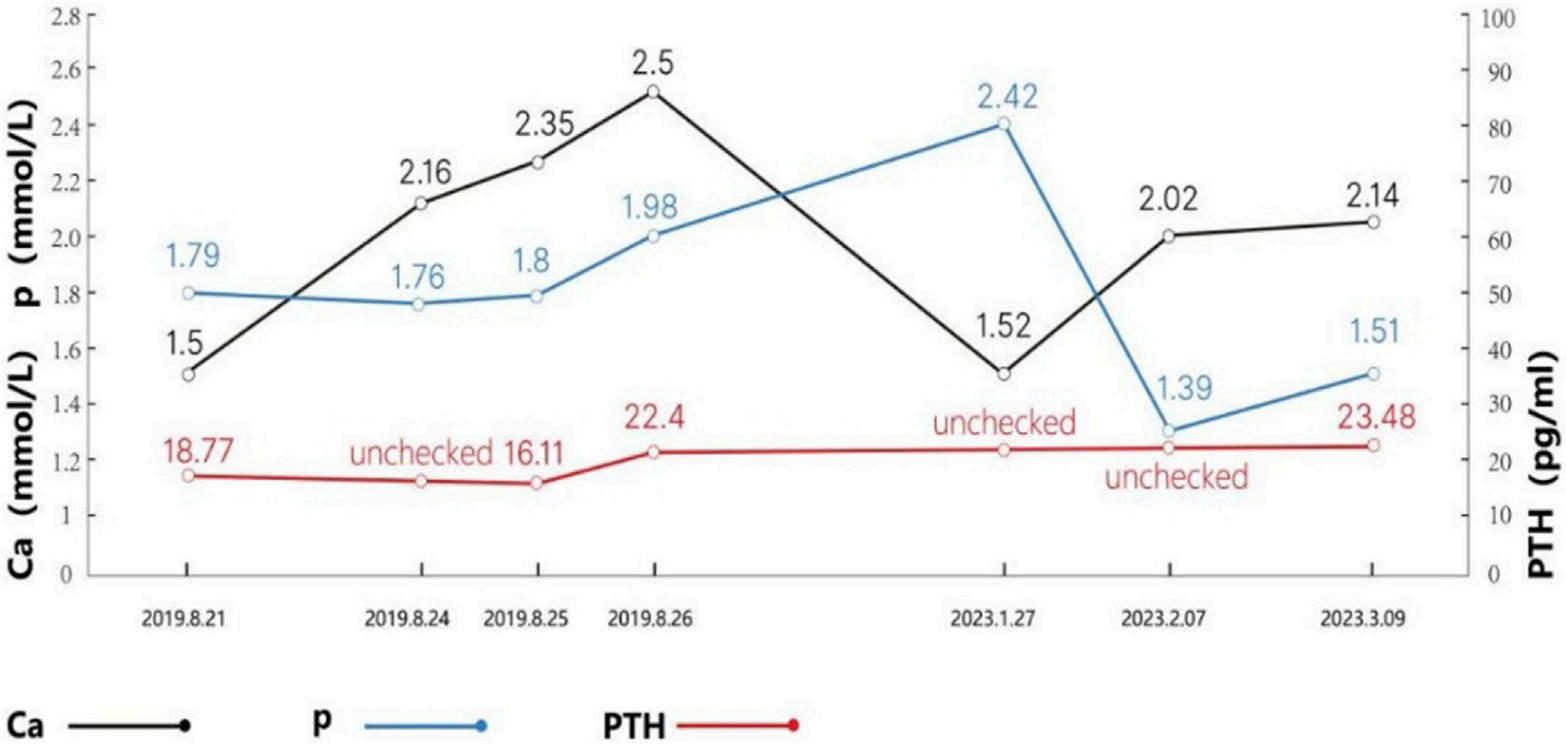
Changes in blood calcium, blood phosphorus, and PTH levels in patients.

After discharge, the patient failed to take medication on time and did not attend regular follow-up appointments. She was examined again on 27 January 2023 to check her blood calcium (1.52 mmol/L) and was given calcium carbonate (1.0 g/day) and calcitriol (osteotriol) (0.25 ug/day). Changes in blood calcium levels, blood phosphorus levels, and PTH levels are shown in Figure 1.

**FIGURE 2:**
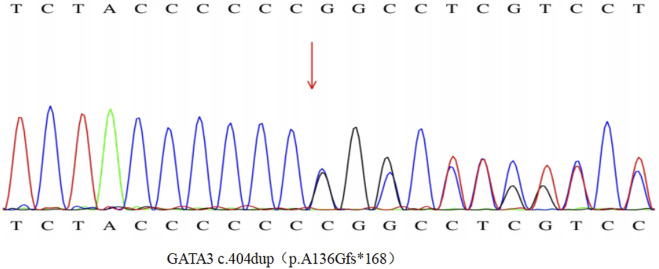
Graph of patients’ genetic test results.

To clarify the diagnosis, with the consent of the patient and her family, a blood sample was taken in February 2023 for hypocalcemia-associated genetic testing, and the results of the genetic examination (Figure 2) showed a heterozygous variant of the *GATA3* gene NM_001002295.2:c.404dup, which causes a change from alanine to glycine at position 136 and triggers a code-shift mutation, culminating in an early termination codon at position 168. This is a pathogenic variant associated with HDRS (OMIM-146255). The remaining family members of this patient did not have similar clinical manifestations, had normal blood calcium levels, and refused genetic testing.

## Discussion segment

The patient was a young woman with a chronic course, insidious onset, and episodes of acute seizures. During the course of the disease, extremely low levels of blood calcium were repeatedly detected, which had not received attention earlier because the symptoms of the patient were not obvious. This time, the patient was hospitalized due to epileptic-like seizures misdiagnosed as epilepsy and was diagnosed with hypoparathyroidism, based on the low blood calcium, low PTH, and high blood phosphorus levels. She was treated with calcium supplementation and active vitamin D, but her blood calcium levels continued to fluctuate during follow-up visits. Combined with the fact that the patient had sensorineural deafness and one side of renal agenesis, HDR syndrome was suspected, which was later confirmed by genetic testing.

The disruption of the zinc finger structural domain of GATA3 is associated with various pathological conditions (Kusakawa et al., 2019). GATA3, a transcription factor characterized by its two zinc-finger domains, ZnF1 and ZnF2, is predominantly expressed in the developing parathyroid glands, inner ear, and kidneys. This protein plays a crucial role in regulating T-lymphocyte differentiation and interacts with TBX21, another transcription factor, via an N-terminal binding domain. Research has indicated that numerous pathogenic variants in GATA3 are situated within or near the DNA-binding domain, downstream of the TBX21 binding domain (Kishi et al., 2020). These variants impair the DNA-binding domain, yet the TBX21-binding domain may persist, leading to a dominant negative impact on GATA3 function from other unaffected alleles. In 2000, it was discovered that heterozygous loss-of-function mutations in GATA3 are responsible for HDR syndrome. Since then, GATA3 mutations have been documented in over 124 cases, with 40% involving shifted-code deletions or insertions, 23% missense mutations, 14% nonsense mutations, 6% splice-site mutations, 1% in-frame deletions or insertions, 15% whole-gene deletions, and 1% whole-gene duplications (Gonçalves et al., 2023). In the present case, the patient was confirmed to have a shifted-code variant through Sanger gene sequencing. Genetic testing revealed that the heterozygous variant NM_001002295.2:c.404dup, originating from exon 3, is the pathogenic variant. This variant not only affects the TBX21 structural domain but may also result in the loss of ZnF1 and ZnF2, potentially leading to aberrant GATA3 function. Consequently, this could manifest as a range of disorders, including hypoparathyroidism, seizures, basal ganglia calcification, intellectual disability, sensorineural deafness, unilateral renal agenesis, and mammary fibroadenoma.

Clinical symptoms caused by different mutation sites in the HDRS gene vary. The heterogeneity of their hypoparathyroidism is obvious, which can range from asymptomatic hypocalcemia to hypocalcemia with obvious clinical symptoms and low or undetectable serum levels of parathyroid hormone; there is also incomplete episodicity in the presence of sensorineural deafness and renal anomalies, whose clinical manifestations, severity, and age of onset vary between individuals (Van Esch et al., 2000).

The age of detection and clinical phenotype of hypoparathyroidism vary widely and can range from subclinical to neonatal hypocalcemic convulsions. In our case, the patient had a significant decrease in blood calcium measured twice without clinical symptoms and later developed epileptiform seizures due to severe hypocalcemia. Dinoi et al. (2024) reported a total of 11 patients carrying the c.404dupC mutation, with a wide range of fluctuating blood calcium levels from 0.83 to 2.04 mmol/L. Among the nine patients with available clinical data, six were asymptomatic (6/9, 66.7%) and three showed epileptiform seizures (3/9, 33.3%). This suggests that there is significant heterogeneity in the clinical presentation of patients with this mutation site, from no clinical signs to epileptiform seizures in the presence of hypocalcemia.

Deafness is the most common symptom of HDRS, present in more than 90% of patients, and is usually the first symptom of HDR syndrome. Studies have shown that GATA3 is required for the functional maturation of hair cells and their innervation in the mouse cochlea (Bardhan et al., 2019). During cochlear development, GATA3 is continuously expressed from the cochlear progenitor site to the mature cochlea. The disruption of GATA3 can lead to abnormal development of the cochlear sensory epithelium and its inaccurate innervation (Xu et al., 2021). In our case, the patient presented with hearing loss 10 years ago, which was detected earlier than hypocalcemia, and the patient also had intellectual disability. Tao et al. (2023) showed that 8.38% of HDRS cases presented with intellectual disability, suggesting that a lack of early management of deafness may be the cause of cognitive impairment. This emphasizes the importance of early recognition and intervention.

Renal anomalies are the most diverse phenotype. It includes congenital renal and urinary tract anomalies, such as renal dysplasia, hypoplasia (41%), cystic kidneys (11%), and vesicoureteral reflux (16%), which may occur bilaterally or unilaterally, as well as abnormalities of renal function, such as proteinuria, uremia, renal tubular acidosis, and renal calcemia. In the kidney, GATA3 is essential for the formation of renal tubes and ureteric buds, which give rise to branches of the ureteric tree and subsequently form collecting ducts. In experimental mice, the deletion of GATA3 leads to the failure of kidney development, and GATA3 deficiency results in the ectopic ureteric outgrowth of the buds and a range of genitourinary malformations (Grigorieva et al., 2019). Although our patient showed a renal agenesis state, she did not show any obvious renal function abnormality for the time being, and she needs to be followed up regularly.

In addition to the typical clinical manifestations, other specific manifestations have been identified in recent years, such as recurrent cerebral infarction, vitamin D deficiency, biliary atresia, female genital tract anomalies, non-autoimmune diabetes mellitus, hypomagnesemia, and congenital heart disease (Juan et al., 2024). Vitamin D deficiency was present in our patient. In addition, some studies in mice have shown that GATA3 is essential for the normal development of mammary tissue and directly regulates luminal cell differentiation, while others have shown that it is integral to estrogen receptor alpha expression and androgen receptor signaling (El-Assaad et al., 2021). The patient had a history of surgical excision for breast fibroadenoma five years prior. During routine follow-up imaging six months ago, multiple persistent hypoechoic nodules were identified bilaterally, which could not be ruled out as being associated with the disease.

Currently, the treatment for patients with HDRS is mainly symptomatic according to the type of symptoms and the severity of the disease (Yang et al., 2022), and intravenous calcium supplementation is mainly used to restore the normal level of blood calcium in a timely manner in patients with hypocalcemic convulsions as a symptom, while patients with chronic hypocalcemia are mainly treated with activated vitamin D and oral calcium supplements. After the blood calcium level increases with intravenous calcium supplementation, active vitamin D and oral calcium supplements can be used for maintenance treatment. There are fewer reports on the therapeutic dosage of calcium and active vitamin D in HDRS patients, and the therapeutic dosage of calcium carbonate at 500 mg BID and osteotriol at 0.25 μg BID was reported in China in one case of the HDRS patient, but the drug adjustment of this patient was not followed up at a later stage (Shu-yao, 2021). Peking Union Medical College Hospital summarized five cases of adolescent HDR medication with blood calcium levels of 1.13–1.87 mmol/L, treated with elemental calcium at 1.0–2.4 g/day and osteotriol at 0.25 ug/day (Yang et al., 2022). A patient carrying the c.404dupC mutation, as reported by Dinoi et al. (2024), was started on treatment with calcium citrate at 1 g/day and osteotriol at 1 ug/day; the doses were gradually increased to 2 g/day and 1.25 ug/day, respectively, after which the patient developed a significant increase in urinary calcium levels; in another family line with a different mutation locus, calcium carbonate at 1.5 g/day and osteotriol at 1 ug/day were applied, and then the dosage was adjusted to 0.5 g/day of calcium carbonate and 0.75 ug/day of osteotriol due to a significantly high level of urinary calcium. Nishimura et al. (2020) reported a case of a patient with HDR who had renal calcinosis and subsequent renal failure due to excessive vitamin D intake. A treatment plan for renal transplantation, but the exact amount of vitamin D intake in this patient was not specified in detail. After living kidney transplantation, the patient’s renal function improved, and the serum calcium level returned to normal, so there was no need for vitamin D supplementation. As in most reports, the patient in this case developed hypercalcemia on the early use of calcium supplements and activated vitamin D, which was normalized after discontinuing osteotriol, and the follow-up review showed that the patient’s calcium and active vitamin D doses were significantly lower than those in other patients with hypoparathyroidism alone.

The prognosis of patients with HDR syndrome depends largely on the severity of renal disease, and many patients have progressive chronic renal failure, of which approximately 9% develop end-stage renal failure and require renal replacement therapy; therefore, early diagnosis and regular testing of renal function are crucial (Ikeuchi et al., 2021).

In summary, this paper reports the diagnosis and treatment process of a patient with HDR, with a view to improving clinicians’ ability to identify and diagnose this disease. For parathyroidism with a younger onset age or earlier onset, especially those with hearing impairment or renal abnormalities, attention should be paid to the screening of hereditary diseases, especially syndromes, and the *GATA3* gene should be screened if necessary for the early diagnosis of HDR syndrome. Once diagnosed, the dosage of calcium and active vitamin D supplements should be individualized and closely monitored to avoid hypercalcemia.

## Data Availability

The original contributions presented in the study are included in the article/supplementary material; further inquiries can be directed to the corresponding authors.
